# Reprocessable thermosets for sustainable three-dimensional printing

**DOI:** 10.1038/s41467-018-04292-8

**Published:** 2018-05-08

**Authors:** Biao Zhang, Kavin Kowsari, Ahmad Serjouei, Martin L. Dunn, Qi Ge

**Affiliations:** 1 Digital Manufacturing and Design Center, Singapore University of Technology and Design, Singapore, 487372 Singapore; 2 College of Engineering and Applied Science, University of Colorado—Denver, Denver, CO 80204 USA; 3 Science and Math Cluster, Singapore University of Technology and Design, Singapore, 487372 Singapore

## Abstract

Among all three-dimensional (3D) printing materials, thermosetting photopolymers claim almost half of the market, and have been widely used in various fields owing to their superior mechanical stability at high temperatures, excellent chemical resistance as well as good compatibility with high-resolution 3D printing technologies. However, once these thermosetting photopolymers form 3D parts through photopolymerization, the covalent networks are permanent and cannot be reprocessed, i.e., reshaped, repaired, or recycled. Here, we report a two-step polymerization strategy to develop 3D printing reprocessable thermosets (3DPRTs) that allow users to reform a printed 3D structure into a new arbitrary shape, repair a broken part by simply 3D printing new material on the damaged site, and recycle unwanted printed parts so the material can be reused for other applications. These 3DPRTs provide a practical solution to address environmental challenges associated with the rapid increase in consumption of 3D printing materials.

## Abstract (web summary)

Thermosetting polymers are widely used in 3D printing owing to their superior mechanical stability, but once they are printed, the highly crosslinked polmyers cannot be reprocessed or repaired. Here the authors demonstrate a two-step polymerization strategy toward 3D printing of reprocessable thermosets.

## Introduction

3D printing technologies are providing new capabilities to fabricate complex 3D geometries, and have become a powerful technique enabling a wide variety of applications, including tissue engineering^1–3^, soft robotics^4–6^, nano-devices^7^, optical engineering^8^, metamaterials^9–11^, and many others^12–14^. Compatibility with UV curing-based 3D printing makes thermosetting photopolymers ideal for printing high-resolution structures at micro-scales^6, 15^, submicro-scales^16^, and even nano-scales^17–19^. However, 3D printed structures formed with the traditional thermosetting photopolymers cannot be reprocessed as the polymer networks are covalent crosslinked^20^. This unprocessable nature, combined with the explosion in 3D printing globally, is leading to vast waste of 3D printing materials with serious environmental implications^21, 22^. Recent advances in the development of dynamic covalent bond (DCB) materials that exploit the reformation and rearrangement of the crosslinked networks to enable reprocessability including self-healing, remolding, and welding offer the possibility of making the thermoset printing materials reprocessable^23, 24^. Shi and co-workers demonstrated the first example of recyclable 3D printing with a DCB-based epoxy. However, the complicated preparation procedure constrains the material to direct-ink-writing 3D printing technology, which limits the printing resolution as well as the product geometric complexity^25^.

**Fig. 1:**
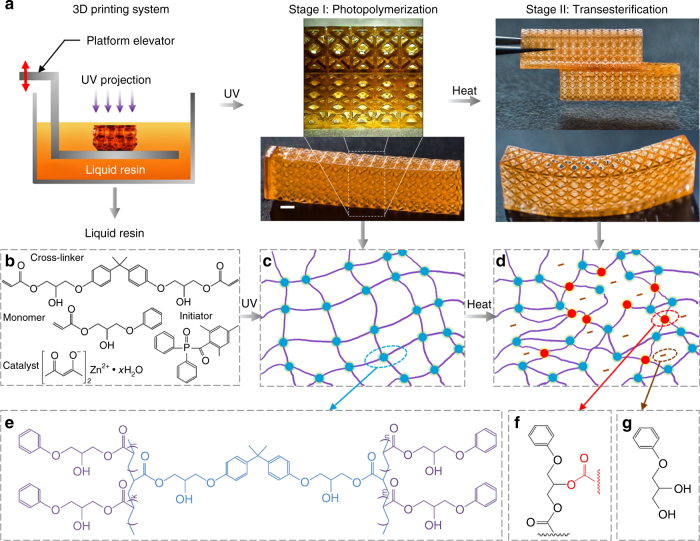
3D printing reprocessable thermosets. **a** General route of 3D printing high-resolution lattice structures with a UV curing-based 3D printing system using the reprocessable thermosetting polymer solution (Stage I). Heating then imparts the reprocessability into the printed structures. Two separate printed lattice structures can be welded together, and a straight lattice structure can be programmed into a bent one (Stage II). Polymer chemistry involved in the two-step polymerization; **b** chemical structures of monomer, crosslinker, initiator, and catalyst in the photopolymer solution; **c** UV curing forms the permanent covalent bonds (blue dots); **d** thermal-triggered transesterification leads to the formation of DCBs (red dots). Chemical structures of the resultant permanent crosslinked network of Stage I (**e**) and DCBs of Stage II after heating (**f**, **g**). Scale bar: 1 mm

Here, we report a two-step polymerization strategy and a simple preparation method to develop a type of 3D printing reprocessable thermosets (3DPRTs) for UV curing-based high-resolution 3D printing. In the developed thermosetting polymer solution, UV reactive acrylate functional groups allow compatibility with UV curing-based 3D printing techniques such as digital light processing (DLP)^15, 26^, mask projection stereolithography^27, 28^, and two-photon lithography^18, 29^, which enables high-resolution 3D printing with complex geometries (Stage I in Fig. 1a). A transesterification reaction between the hydroxyl and ester functional groups upon heating^30, 31^ then forms DCBs that impart reprocessability into the printed structures (Stage II in Fig. 1a). As illustrated in Fig. 1b, we prepared the polymer solution by mixing 2-hydroxy-3-phenoxypropyl acrylate as monomer, bisphenol A glycerolate (1 glycerol/phenol) diacrylate as crosslinker, diphenyl(2,4,6-trimethylbenzoly) phosphine oxide as photo initiator to trigger the UV polymerization, and zinc acetylacetone hydrate as catalyst to accelerate the transesterification reaction (see Methods about the details). During 3D printing, patterned UV irradiation provided via a digital micromirror device stimulates localized photopolymerization by opening the double bonds on the acrylate functional groups on both the monomer and crosslinker to form permanent covalent bonds (blue dots in Fig. 1c and the detailed chemical structure in Fig. 1e; see the measurement of UV polymerization in the Supplementary Note 1 and Supplementary Fig. 1) and solidify the liquid polymer solution into the corresponding solid pattern. Layer-by-layer solidification continues until the fabrication of an entire 3D structure is complete^32^ (see Methods for details). Subsequent heating to an elevated temperature (for example, 180 °C) where the transesterification between the ester and hydroxyl groups proceeds at a fast rate, results in the formation of DCBs (red dots in Fig. 1d) within a few hours. Formation of dynamic bonds evolves simultaneous breaking and reconnecting between the ester and hydroxyl groups, which means the total number of the covalent bonds maintains the same (see Fig. 1f, g, Supplementary Note 2 and Supplementary Fig. 2), while the crosslinking density continues increasing until the reaction reaches dynamic equilibrium^31^ (see Supplementary Note 3 and Supplementary Fig. 3).

## Results

### Reshapability of 3DPRTs

**Fig. 2:**
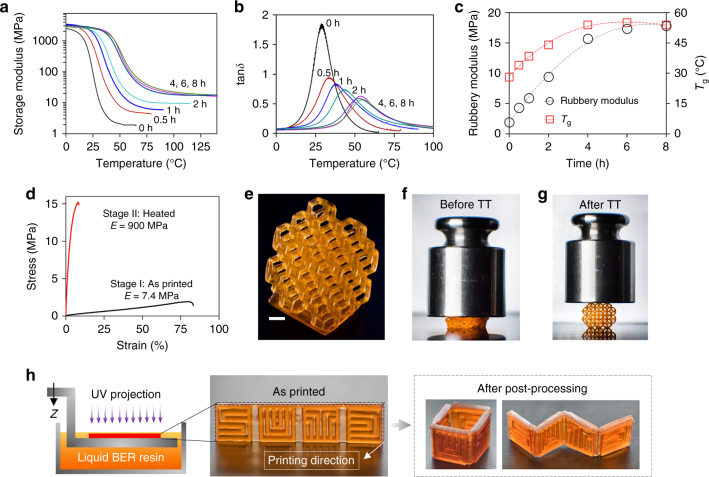
Reshapability of 3D printing reprocessable thermosets. **a**–**c** Dynamic mechanical analysis (DMA) tests to investigate the effect of thermal treatment on mechanical properties of samples printed with reprocessable thermosets. **d** Uniaxial tensile tests of printed samples before and after the 4 h thermal treatment at 180 °C. Demonstration of stiffness change of a printed Kelvin foam (**e**), before (**f**), and after (**g**) thermal treatment (TT) at 160 °C for 12 h. **h** Demonstration of combining 3D printing with traditional thermoforming to expand capability and reduce time of manufacturing. Scale bar: 1 mm

To study the effect of the thermal treatment on the mechanical properties, we conducted dynamic mechanical analysis (DMA) tests with 3D printed strip samples that were thermally treated at 180 °C for 0, 0.5, 1, 2, 4, 6, and 8 h, respectively (see Methods for details). Figure 2a and b shows the storage modulus and tan*δ* vs. temperature, where the storage modulus describes the elastic response of the material and the peak of tan*δ* indicates the glass transition temperature (*T*_g_). The increase of the thermal treatment time from 0 to 4 h (Fig. 2a) results in a gradual increase in the rubbery modulus (the lower modulus plateau at high temperatures) from ~ 2 to ~20 MPa (Fig. 2c shows the relation between the thermal treatment duration and rubbery modulus), which suggests an increase in DCBs during the bond exchange reactions (BERs). After the 4 h thermal treatment, the DCBs reach a dynamic equilibrium beyond which no apparent increase in rubbery modulus is observed (Fig. 2a, c). As shown in Fig. 2b and c, the increase in DCBs does not only leads to the rise of rubbery modulus but also shifts the peak of tan*δ* to a higher temperature as the introduction of additional crosslinks restricts segmental chain mobility, and therefore results in the increase in *T*_g_^33–35^. The increased *T*_g_ stretches the glassy state to a high temperature region and converts the compliant material at room temperature with Young’s modulus of 7.4 MPa into a stiff one with Young’s modulus of ~900 MPa (Fig. 2d, see Methods for details). In Fig. 2e–g, we demonstrate this mechanical property change by first placing a 100 g weight on a 3D printed Kelvin foam (Fig. 2e) without the thermal treatment. The untreated structure cannot support the weight and is deformed severely (Fig. 2f). After the 4 h thermal treatment, the structure stiffness increases significantly and enables it to support the 100 g weight without any apparent deformation (Fig. 2g). This significant stiffness increase upon the heat treatment facilitates the reshapability of the 3D printed structures. We can exploit this property to combine 3D printing with traditional manufacturing methods, such as molding, pressing, and thermoforming, to increase manufacturing capabilities and decrease manufacturing time. We demonstrate this concept in Fig. 2h. Instead of directly 3D printing standing structures, we printed a thin strip with the letters SUTD in the thickness direction, which minimizes the number of layers and thus the printing time. The strip was then thermoformed into 3D cubic and wavy shapes that would require much longer printing time if we print them directly.

### Repairability of the 3DPRTs

**Fig. 3:**
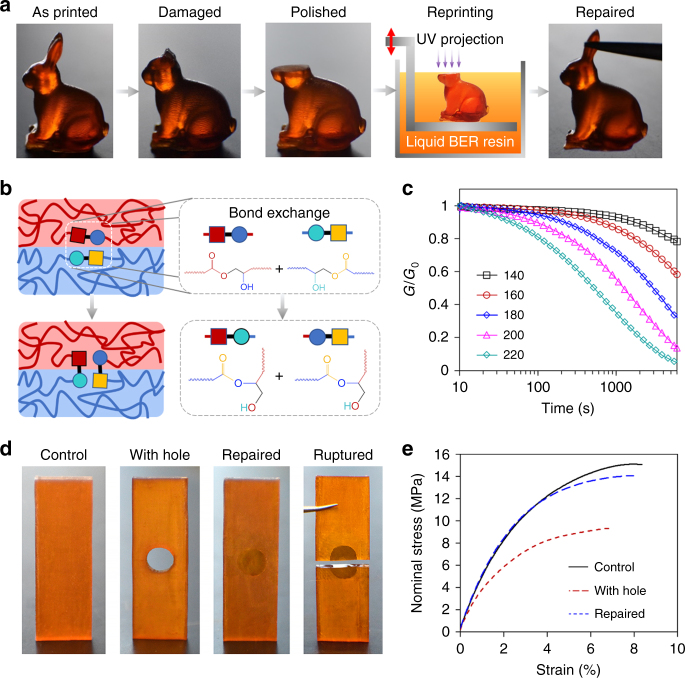
Repairability of the 3D printing reprocessable thermosets. **a** Demonstration of the ability of the material to repair flawed printed structures: surgery on a damaged rabbit. **b** Illustration of the repair process due to the BER. **c** The temperature effect on stress relaxation of printed samples. **d**, **e** Uniaxial tensile tests to examine the repair performance. **d** Photos of the control sample, a sample printed with a hole, a repaired sample, and a ruptured sample after repair. **e** Comparison of the nominal stress (force divided by cross-section area of control sample) vs. strain for the samples in **d**

With conventional thermosetting 3D printing materials, once a printed structure is damaged, it cannot be repaired as the chemically crosslinked networks are permanently destroyed. 3DPRTs change this view as the DCBs make the printed structures repairable through thermally activated self-healing^36–38^. In Fig. 3a, we repair a 3D printed rabbit that has lost its ears, by first polishing the damage site to achieve a flat surface, and then conducting the 3D printing new material on the polished surface to rebuild the missing part of the rabbit. After printing, the rabbit was heated to 180 °C for 4 h to regain the mechanical performance. Figure 3b illustrates the repair mechanism based on the heat-triggered BERs where the dynamic crosslinking points break up after being attacked by the adjacent hydroxyl functional groups, and later reform new dynamic crosslinking points by connecting with the adjacent ester functional groups. The topological rearrangement of the macromolecular networks builds DCBs across the interface, and eventually bonds the original part with the rebuilt part, resulting in a homogeneous repaired solid. Macroscopically, breaking of the dynamic crosslinks during the BER results in stress relaxation. In Fig. 3c, we investigate the temperature effect on the stress relaxation (see Methods for details). At 220 °C, more than 80% of the stress is relaxed within 40 min, while at 140 °C more than 90% of the stress is unrelaxed. This indicates the BERs are strongly temperature dependent, and the functional groups involved in BERs are more active at higher temperatures. The temperature-dependent characteristic relaxation time τ* can be expressed by the Arrhenius equation with the activation energy *E*_a_ = 77 kJ mol^−1^, which is similar to other transesterification reaction based polyester networks^30, 31^ (see Supplementary Note 4 and Supplementary Fig. 4). During BERs, dynamic equilibrium of the breaking-reforming process renders the total number crosslinks constant, which ensures that the repaired structure largely restores the mechanical performance of the original one. To examine this point, we printed a strip with a circular hole to simulate a mechanical flaw (Fig. 3d). We repaired the strip by (i) filling the hole with the reprocessable thermoset solution, (ii) irradiating it with UV light, and (iii) heating it (see Methods for details). Figure 3e compares the mechanical performance in uniaxial tensile tests of an unflawed control sample, the flawed sample with a hole, and the repaired sample. The repaired sample recovers ~100% of the stiffness, and 93% of strength indicating the healing progress robustly bonds the separate parts and restores the mechanical performance (see Methods for details). In addition, the fact that the fracture boundary passes through the repaired circle rather than following the circular boundary (Fig. 3d) shows that robustness of the repair process. In contrast, we attempted the same repair approach with a flawed strip sample printed with a conventional thermoset 3D printing material, VeroClear (Stratasys, MN, USA) (see Supplementary Note 5 for details). However, as shown in Supplementary Figs. 5 and  6, after repaired, the improvement in the mechanical performance is limited, and the fracture boundary follows the repaired circular boundary. This indicates that any improvement derives from mechanical blocking of the solid material in the circular hole, and not the creation of new covalent bonds between the newly deposited and existing materials, and shows the inability to repair conventional 3D printed thermosets. In addition, comparing Fig. 3e with Supplementary Fig. 5, it is noted that the sample printed with 3DPRT has inferior mechanical properties compared to the conventional 3D printable thermoset material (VeroClear), which limits the potential applications of the 3DPRT^39^. To enhance the mechanical performance of the 3DPRT in both strength and toughness, we suggest to introduce non-covalent sacrificial bonds^40–42^ or nanoparticle and fibers^43–45^ into the 3DPRT network.

### Recyclability of 3DPRTs

**Fig. 4:**
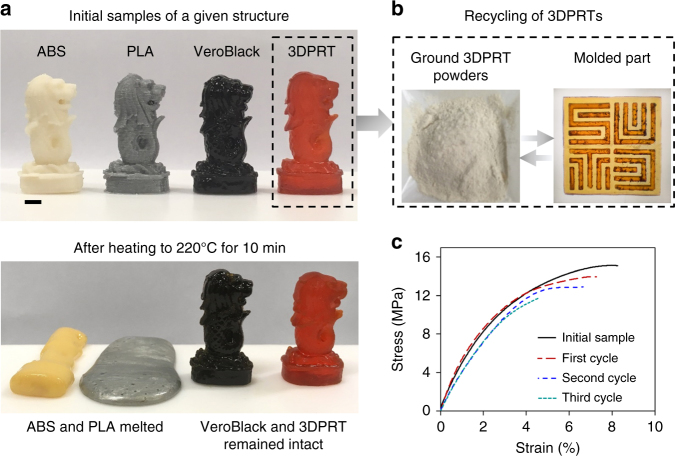
Recyclability of 3D printing reprocessable thermosets. **a** Stability comparison of a printed structure with 3DPRT and structures printed with commercial available thermoplastics (PLA and ABS) and thermoset (Vero-black) at high temperature (220 °C). **b** Demonstration of recycling of a structure printed with 3DPRT. **c** Uniaxial tensile tests to examine the mechanical repeatability of the recycled 3DPRTs. Scale bar: 5 mm

In addition, compared to thermoplastic 3D printing materials such as Acrylonitrile-Butadiene Styrene (ABS) and Poly Lactic Acid (PLA) which melt at high temperatures, thermosetting 3D printing materials are dimensionally stable at high temperatures due to the chemically crosslinked networks (Fig. 4a). However, these chemically crosslinked networks also make recycling of these thermosetting 3D printing materials technically challenging and/or cost ineffective. The 3DPRTs developed here exploit BERs to realize recyclability of thermosetting 3D printing materials, which offers a promising contribution to the environmental challenges of polymer recycling. As shown in Fig. 4b, we recycled the 3DPRTs by grinding the printed structure into powders. The powders were then poured into a mold with the SUTD pattern. After the thermal treatment, a thermosetting sheet with SUTD letters was formed due to the BER (Fig. 4b). This recycling process is repeatable. Figure 4c shows uniaxial tensile testing results for repeatedly recycled samples and despite slight mechanical degradation after each recycling treatment, the overall mechanical performance of the recycled sample is reasonably good.

## Discussion

In summary, we developed a 3DPRT material system using a two-step polymerization strategy. The reprocessable thermosets impart reshapeability, repairability, and recyclability into 3D printed structures, and can contribute to alleviate environmental challenges associated with the continuous increase in consumption of 3D printing materials.

## Methods

### Material preparation

45 g of 2-hydroxy-3-phenoxypropyl acrylate and 3.0 g of the catalyst (zinc acetylacetone hydrate, Zn(acac)_2_) were first added in a 100 ml bottle. The solutions were mixed at around 70 °C until the good solubility of the catalyst was achieved. Then, diphenyl(2,4,6-trimethylbenzoly) phosphine oxide (1.0 g) as initiator was added at room temperature. After the miscibility occurred, 5.0 g of bisphenol A glycerolate (1 glycerol/phenol) diacrylate used as crosslinker was added into the systems. Finally, the weight ratio of monomer and crosslinker is 9:1, and the molar ratio of ester groups and hydroxyl groups is 1:1. The concentration of catalyst is 5 mol% to the OH groups. The amount of the initiator is 2 wt.% of the total weight of monomer and crosslinker. For 3D printing, Sudan I (0.02 wt.% of the total weight) was added as photo absorber. The 3DPRT polymer solutions were stored in amber laboratory bottles at 5 °C. The polymer solutions are stable for more 2 months without any gelation and precipitation, and can be used directly for 3D printing. All chemicals were purchased from Sigma-Aldrich (Singapore) and used as received.

### 3D printing

We used the prepared 3DPRTs to print 3D structures on a custom DLP-based high-resolution 3D printing system^26^. The exposure time is 2 s for each layer with the given thickness of 50 μm. After printing, the unreacted monomers and crosslinkers on the surface of the printed 3D structures were removed by using a rubber suction bulb. Finally, the structures were post-cured in a UV oven (UVP, Ultraviolet Crosslinkers, Upland, CA, USA). According to the results shown in Supplementary Fig. 1, the post-curing time is required to be more than 5 min. ABS and PLA Merlion samples in Fig. 4a were printed using a commercial Fused Deposition Modeling-based 3D printer (Fortus 450MC, Stratasys, USA). The Veroblack Merlion sample in Fig. 4a was printed using a commercial Polyjet 3D printer (Stratasys J750, MN, USA).

### Thermal treatment

Thermal treatments were conducted by placing UV cured samples in a universal heating oven (Memmert Oven U, Germany) at a set temperature for a set period of time.

### Material characterizations

Fourier Transform Infrared Spectroscopy (FT-IR) tests were conducted on a VERTEX 70 FT-IR spectrometer (Bruker, Germany) using Attenuated Total Reflection mode with a Magna-IR Nicolet 550 collecting 32 scans from 400 to 4000 cm^−1^.

DMA tester (Q800 DMA, TA Instruments) was used to characterize the thermomechanical properties in the tension film mode. Samples with the dimension of 15 mm × 5 mm × 0.5 mm were tested at a frequency of 1 Hz and an amplitude of 5 μm. The temperature was first equilibrated at −20 °C for 5 min, and then increased at a heating rate of 3 °C/min. The glass transition temperatures (*T*_g_) were determined from the peak of the tan*δ*.

Stress relaxation tests were performed on the DMA tester (Q800 DMA, TA Instruments) using 3-point bending mode on rectangular samples (35 mm × 9.0 mm × 1.8 mm) made by curing the 3DPRT polymer solution in a Teflon mold. After loading, the sample was equilibrated at the specified temperature (from 140 to 220 °C) for 10 min. A constant deflection of 0.018 mm was applied to monitor the stress relaxation for 100 min.

Uniaxial tensile tests were performed using an Instron Machine mounted with a 1 kN cell to determine the mechanical performance of the materials at room temperature. The strain rate was set as 10 mm/min. The sample size was 30 mm × 10 mm × 1.5 mm. The distance between the two grips was 15 mm. Young’s modulus (*E*) value was obtained from the initial slope of the stress-strain curve.

### Self-repairing experiments

The control sample and the sample with a hole were directly printed on the custom printer with the 3DPRT polymer solution. The dimension of the printed strip samples was 30 mm in length, 10 mm in width, and 1.5 mm in thickness. For the samples printed with a hole, the diameter of the hole was 5 mm. All the printed samples were heated at 180 °C for 4 h. To repair the sample printed with a hole, we first filled the hole with the 3DPRT polymer solution, and then applied the UV (365 nm) irradiation for 10 min and thermal treatment at 180 °C for 4 h. The mechanical tests were performed on the Instron Machine.

### Recycling

Samples obtained using the general procedure for the preparation of BER networks were grinded into fine powders and sandwiched between two foil-coated metal plates. This assembly was heated under a hydraulic press with 500 MPa pressure at 220 °C for 2 h. After cooling to room temperature, the sample was demolded to yield defect-free materials. The obtained cylindrical samples were trimmed into rectangular samples for uniaxial tensile experiments by Waterjet. This same method was repeated three times for reprocessing tests. The SUTD logo was molded by using a custom-made mold.

### Data availability

The data that support the findings of this study are available from the corresponding author on request.

## Electronic supplementary material

Supplementary Information
