# Supplementary material for: Reprocessable thermosets for sustainable three-dimensional printing
Source: Nat Commun. 2018 May 8;9:1831. doi: 10.1038/s41467-018-04292-8 (PMC5940800; doi:10.1038/s41467-018-04292-8)
Supplement: Supplementary file 1 — Supplementary Information [file 41467_2018_4292_MOESM1_ESM.pdf]

Supplementary Information for Reprocessable Thermosets for Sustainable Three-dimensional Printing

Biao Zhang[1], Kavin Kowsari[1], Ahmad Serjouei[1], Martin L. Dunn[1,2], and Qi Ge[1,3]

---

[1] Digital Manufacturing and Design Center, Singapore University of Technology and Design, 487372, Singapore. [2] College of Engineering and Applied Science, University of Colorado, Denver, Denver, Colorado 80204. [3] Science and Math Cluster, Singapore University of Technology and Design, 487372, Singapore. Correspondence and requests for materials should be addressed to M. L. D. (martin.dunn@ucdenver.edu) and Q.G. (ge_qi@sutd.edu.sg).

Supplementary Note 1: UV polymerization measured by FT-IR spectrum

The prepared 3D printing reprocessable thermoset (3DPRT) polymer solution was poured into a home-made Teflon mold (50 × 50 × 2 mm^3) and covered with a transparent Teflon film. Then the mold was placed into a UV oven (UVP, Ultraviolet Crosslinkers, Upland, CA, USA) and cured for 5 min. Fourier Transform Infrared Spectroscopy (FT-IR) tests were conducted on a VERTEX 70 FT-IR spectrometer (Bruker, Germany) using Attenuated Total Reflection (ATR) mode with a Magna-IR Nicolet 550 collecting 32 scans from 400 to 4000 cm^{-1}.

Supplementary Fig. 1 shows the FT-IR spectra of materials before and after UV curing process. Based on the disappearance of the absorption peaks of methylene group vibrations at 1040 cm^{-1} (assigned to out-of-plane bending mode of the =C-H unit), we can conclude that nearly all the monomers and crosslinkers are consumed after curing for 5 min under UV oven. This provides a guideline for the post-curing process of 3D printed structures.

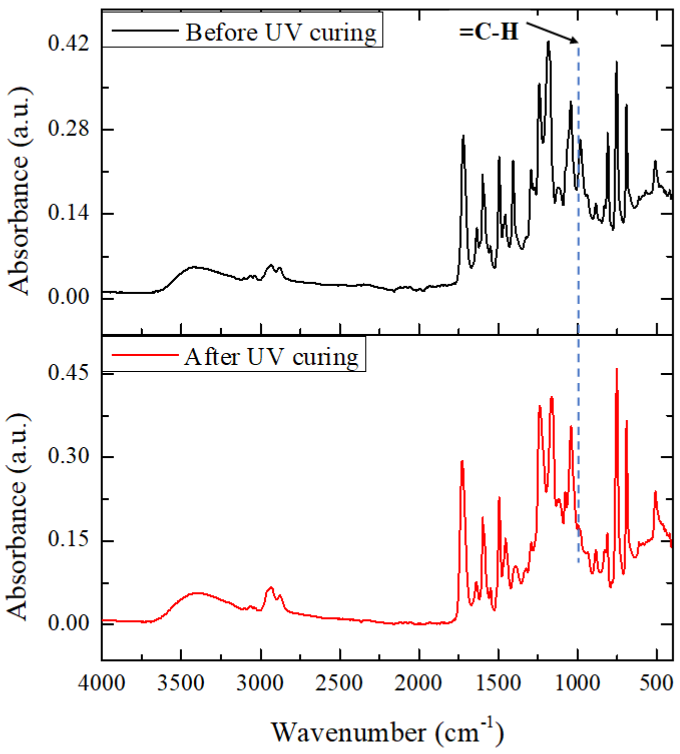

Supplementary Figure 1. FT-IR spectra of the prepared 3D printing reprocessable thermoset (3DPRT) polymer solution (up) and its corresponding material (down) obtained by UV polymerization for 5 minutes under UV irradiation (365 nm).

Supplementary Note 2: Transesterification measured by FT-IR spectrum

Fourier Transform Infrared Spectroscopy (FT-IR) was conducted to monitor the thermal treatment process (180 °C, from 0 h to 8 h). Supplementary Fig. 2 shows there is no apparent change during the thermal treatment, showing that there is a dynamic equilibrium between esters and hydroxyls groups during transesterification, and no new functional groups appear.

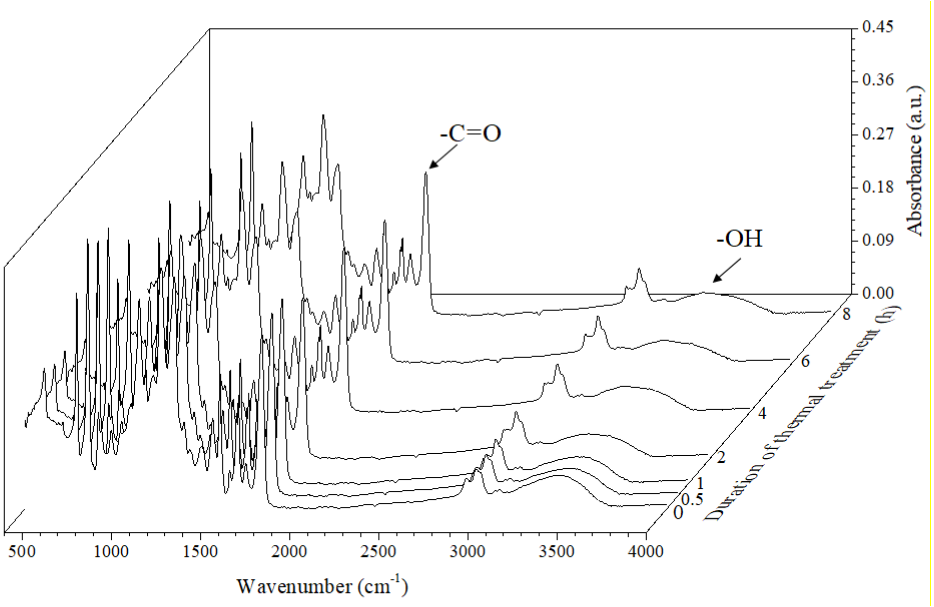

Supplementary Figure 2. FT-IR measurements during thermal treatment of 3D printing reprocessable thermosets at 180 °C from 0 h to 8 h.

Supplementary Note 3: Scheme for transesterification during thermal treatment

Supplementary Fig. 3 shows the dynamic transesterification during the thermal treatment. In our case, a lightly crosslinked network was formed during the 3D printing step (UV curing) due to the contribution of bifunctional acrylate (i.e. bisphenol A glycerolate (1 glycerol/phenol) diacrylate). Upon heating to a high temperature (e.g. 180 °C), the transesterification takes place between the ester and hydroxyl groups, which leads to the appearance of numerous products.[1] Especially, new crosslink points appear during the bond exchange reaction, which lead to the materials much stiffer. Due to the large amount of the ester and hydroxyl groups which are from monomers and crosslinkers, the crosslinker density of the polymer network continues increasing until the reaction reaches the dynamic equilibrium where the concentrations of all species are equilibrated. [1]

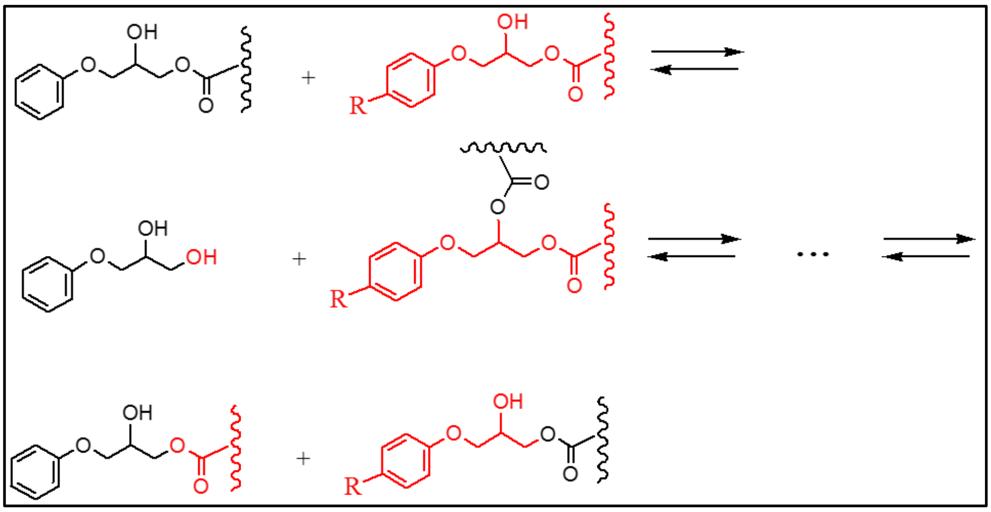

Supplementary Figure 3. Illustration of the dynamic transesterification during the thermal treatment.

Supplementary Note 4: Determination of the activation energy of the transesterification reaction

The relaxation modulus $G(t)$ is a time dependent function at different temperatures and can be expressed as

$$G(t) = G_0 \exp\left(-t/\tau^*\right), \tag{1}$$

where G_0 is the initial modulus at $t = 0$ and τ^* is the characteristic relaxation time which can be obtained by fitting relaxation modulus *vs*. time plots in Fig. 3c using Supplementary Equation 1.

In Supplementary Fig. 4, we plotted the characteristic relaxation time τ^* as a function of the inverse temperature, and fitted this relation using the Arrhenius equation:

$$\ln \tau^*(T) = \ln \tau_0 + E_a/RT \,, \tag{2}$$

where τ_0 is the characteristic relaxation time at the infinite temperature, E_a is the activation energy of the transesterification reaction ($J \cdot mol^{-1}$), R is the universal gas constant ($J \cdot mol^{-1} \cdot K^{-1}$) and T is the

temperature at which the stress relaxation experiment was performed ranging from 453.15 to 493.15 K. The obtained the activation energy E_a is 77 kJ·mol^{-1}.

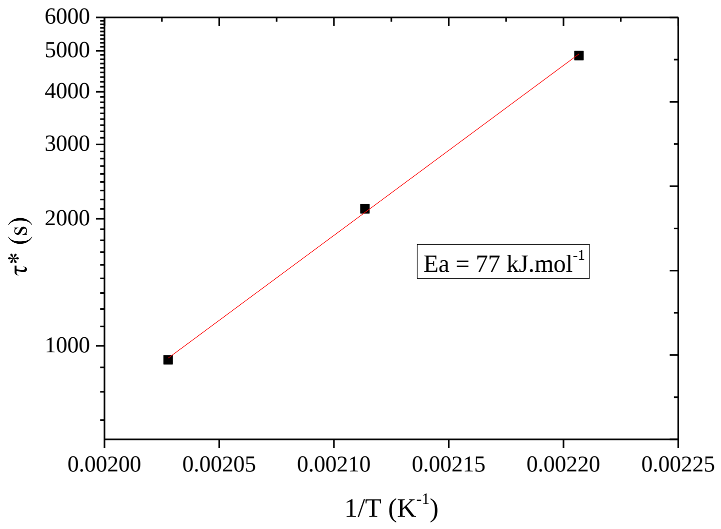

Supplementary Figure 4. Evolution of characteristic relaxation times *vs.* the reciprocal temperature for the 3DPRT materials.

Supplementary Note 5: Comparison of repair experiments

As a comparison, we attempted the same repair approach with a flawed strip sample printed with a conventional thermoset 3D printing material, VeroClear (Stratasys, MN, USA). The control sample, and the sample with a hole were directly printed by using Polyjet (Stratasys, MN, USA) with VeroClear solution. The dimensions of the printed strip samples are the same with the samples printed by 3DPRT polymer solution, which are 30 mm in length, 10 mm in width and 1.5 mm in thickness. For the samples printed with a hole, the diameter of the hole is 5 mm. All the printed samples were heated at 180 °C for 4 h. To repair the sample printed with a hole, we first filled the hole with the VeroClear solution, and then applied the UV (365 nm) irradiation for 10 min and thermal treatment at 180 °C for 4 hours. The mechanical tests were performed on the Instron Machine. As shown in

Supplementary Fig. 5, the mechanical performance of the “repaired” sample was slightly higher than the sample with a hole, due to the physical interactions, but apparently lower than the control sample because of the lack of covalent bonds. The snapshot of the broken “repaired” VeroClear sample illustrates the weak connections between the hole samples with the filled resins (See Supplementary Fig. 6).

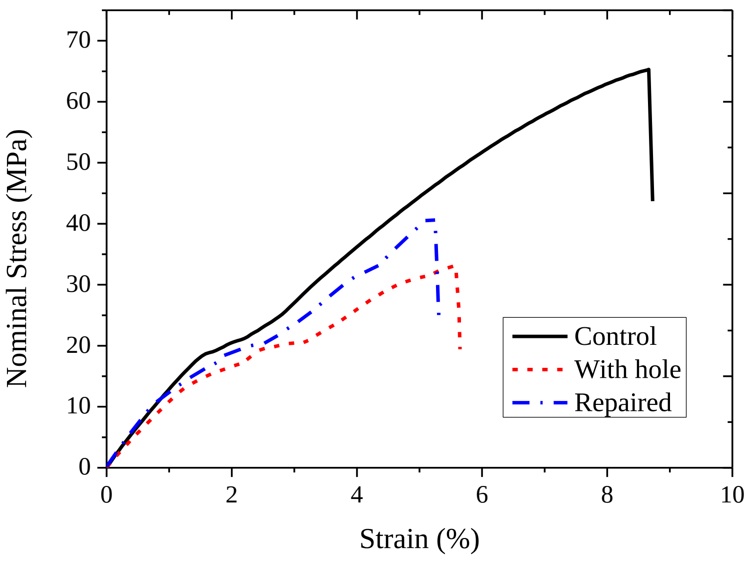

Supplementary Figure 5. Self-repair test results of VeroClear based samples.

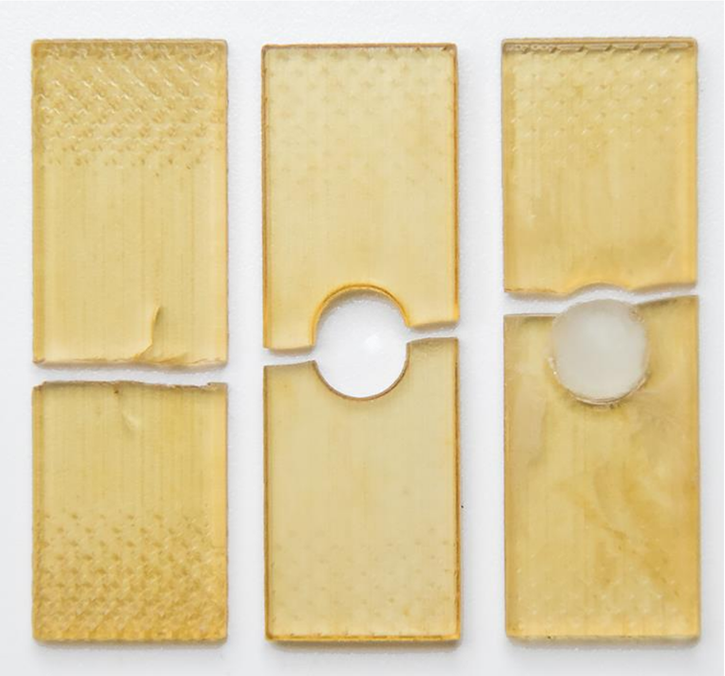

Supplementary Figure 6. Snapshots of broken VeroClear samples (From left to right: the control sample, the sample with a hole and the filled sample).

Reference

1. Capelot M., Montarnal D., Tournilhac F., and Leibler L. Metal-Catalyzed Transesterification for Healing and Assembling of Thermosets. *J. Am. Chem. Soc.* **134**, 7664−7667 (2012).
